# Investigation of Interactive Effects on Water Flow and Solute Transport in Sandy Loam Soil Using Time Domain Reflectometry

**DOI:** 10.3390/s120709749

**Published:** 2012-07-18

**Authors:** Hasan Merdun

**Affiliations:** Department of Environmental Engineering, Faculty of Engineering, Akdeniz University, 07058 Antalya, Turkey; E-Mail: merdun@alumni.clemson.edu; Tel.: +90-242-3106-358; Fax: +90-242-3106-306

**Keywords:** vadose zone, field, soil properties, application rate, permittivity, moisture sensing, modeling

## Abstract

Surface-applied chemicals move through the unsaturated zone with complex flow and transport processes due to soil heterogeneity and reach the saturated zone, resulting in groundwater contamination. Such complex processes need to be studied by advanced measurement and modeling techniques to protect soil and water resources from contamination. In this study, the interactive effects of factors like soil structure, initial soil water content (SWC), and application rate on preferential flow and transport were studied in a sandy loam field soil using measurement (by time domain reflectometry (TDR)) and modeling (by MACRO and VS2DTI) techniques. In addition, statistical analyses were performed to compare the means of the measured and modeled SWC and EC, and solute transport parameters (pore water velocity and dispersion coefficient) in 12 treatments. Research results showed that even though the effects of soil structural conditions on water and solute transport were not so clear, the applied solution moved lower depths in the profiles of wet *versus* dry initial SWC and high application rate *versus* low application rates. The effects of soil structure and initial SWC on water and solute movement could be differentiated under the interactive conditions, but the effects of the application rates were difficult to differentiate under different soil structural and initial SWC conditions. Modeling results showed that MACRO had somewhat better performance than VS2DTI in the estimation of SWC and EC with space and time, but overall both models had relatively low performances. The means of SWC, EC, and solute transport parameters of the 12 treatments were divided into some groups based on the statistical analyses, indicating different flow and transport characteristics or a certain degree nonuniform or preferential flow and transport in the soil. Conducting field experiments with more interactive factors and applying the models with different approaches may allow better understanding of flow and transport processes in addition to the simulations of them in the unsaturated zone.

## 1. Introduction

Flow and transport processes in the unsaturated or vadose zone control the time and degree of groundwater pollution because the surface-applied chemicals need to pass this zone first to be able to reach groundwater. The factors or processes taking place in this zone are numerous and complex due to the soil heterogeneity. Advanced experimental or modeling tools are required to understand the mechanisms of such complex flow and transport processes. Such tools allow us to develop good management practices to protect soil and groundwater from contamination because of the land-applied chemicals, like agricultural fertilizers and pesticides.

Water and solutes can move through the vadose zone along preferred pathways, such as soil cracks, worm holes, and root channels [1,2]. This non-equilibrium phenomenon, known as preferential flow, causes contaminants to reach great depths through these large openings in the soil in relatively short times [3–5]. Unlike uniform flow, preferential flow causes irregular wetting of the soil profile due to water moving faster in certain parts of the soil profile than the other parts [6–8]. Three main types of preferential flow processes are: macropore flow through cracks, worm holes, and root channels in structured soils [1], unstable finger flow [9], and funnel flow [10,11]. The latter two processes occur in the soil matrix, predominantly in sandy textured soils. Therefore, preferential flow can occur in almost all types of soils caused by heterogeneities at scales ranging from the single pore to the pedon [12]. The extent of preferential flow and transport depends on factors such as soil texture, structure, initial soil water content (SWC), and application rate [13,14]. Even though flow is uniform in deep and uniform sandy soils, finger flow is observed in layered sandy soils having different particle sizes [15]. Infiltration is higher in non-tilled or undisturbed soils than in tilled or disturbed soils [13,16,17]. There is no consensus among the researchers about the effect of initial SWC on the preferential flow and transport [18–21]. Chloride-tagged water with lower application rates in sandy and sandy loam soils reach deeper depths than in clayey soil [22,23]. They reported that high rainfall intensity after herbicides application caused considerable amount of them to leach deeper depths in a sandy loam soil. In the case of higher application rate, the more preferential flow was observed [24]. These studies show that the effects of individual factors affecting preferential flow and transport need to be further investigated. The interaction of the controlling parameters may help better understanding of these processes.

Since the transport of water and solute in the vadose zone is not uniform due to soil heterogeneity, classical models using the Richards Equation do not produce acceptable results [1,25–28]. Therefore, different approaches are required for reliable simulations of flow and transport in this zone. A variety of approaches are available for modeling preferential flow and transport in the vadose zone like dual-porosity [29], dual-permeability [30], multi-porosity, and multi-permeability models [31]. In the dual-porosity and dual-permeability modeling approaches, the total soil porosity consists of two regions, macropore and micropores. Water flows through only macropores in a dual-porosity model, but it flows through both regions in a dual-permeability model. Accurate or reliable measurement of water flow and solute transport in the vadose zone is a key factor in better understanding the mechanisms of flow and transport processes and modeling these processes.

Enabling to measure both SWC [32] and soil bulk electrical conductivity, EC [33] simultaneously makes time domain reflectometry (TDR) a valuable tool for studying the transport processes of water and chemical movement in the unsaturated zone in laboratory or field under steady-state or transient conditions. The main advantage of the TDR over other traditional measurement techniques like soil coring and suction samplers is the acquisition of reliable, accurate, rapid, continuous, automatic, non-destructive, and real-time spatio-temporal field measurements of water flow and solute transport parameters [34,35]. A large set of data can easily be collected in the field by replicating the TDR probes through multiplexing [36–38].

Studies focusing on the understanding of the mechanisms of preferential flow and transport processes are still being intensively conducted. Even though several studies have been conducted on the investigation of individual factors like soil texture, structure, initial SWC, and application rate on preferential flow and transport in the laboratory conditions [39–42], the collective effects of these factors on preferential flow and transport in a sandy loam field soil using TDR have not been thoroughly investigated. More importantly, the interactive effects of these factors on preferential flow and transport have not been studied using TDR. Using TDR under field conditions will be very helpful in finding more realistic solutions to water and solute transport problems. In addition, modeling studies on preferential flow and transport for heterogeneous field soils are relatively limited, which may be due to the difficulty in collecting reliable data. The better understanding of the mechanisms of preferential flow and transport through experimental and modeling studies will help to improve the effective soil and water resources management; as a result, protecting soil and groundwater from the negative impacts of agricultural chemicals.

The objectives of this study are to investigate the individual and interactive effects of factors like soil structure, initial SWC, and application rate on the extent of preferential flow and transport in a sandy loam field soil by using TDR to measure SWC and EC; modeling the TDR-measured SWC and EC data by MACRO and VS2DTI; comparing the treatment means of SWC and EC, and solute transport parameters (pore water velocity and dispersion coefficient), obtained by fitting the TDR measured breakthrough curve (BTC) data to the one-dimensional convection-dispersion equation (CDE) in the CXTFIT program, by means of statistical analyses.

## 2. Materials and Methods

### 2.1. Study Area

Field experiments were conducted on a sandy loam soil located about 15 km far from the center of Kahramanmaraş City in Turkey (31°55′28″E and 41°54′54″N) between August 11 and September 17, 2007. The area was located on a private farm, where mostly vegetables and corn were grown, but it was not planted in the year experiments were conducted. The study area is under the Mediterranean climatic conditions, characterized mainly by hot and dry summers and warm and rainy winters. The mean annual temperature was 16.3 °C and mean annual rainfall was 708.1 mm. The mean highest evaporation was 333.3 mm.

### 2.2. Soil Properties and Application Rates

Some physical and chemical properties (Table 1) of the experimental field soil were determined before the experiment by collecting the disturbed and undisturbed soil samples from seven depths of the soil profile with three replicates. All samples were analyzed in accordance with [43,44]. For the analyses of bulk density and saturated hydraulic conductivity the undisturbed soil samples were used, whereas the other analyses were conducted on the disturbed soil samples. Bulk density was determined by using standard 100 cm^3^ cylinders. For saturated hydraulic conductivity determination, the “Constant Head Permeameter” method [44] was used. The disturbed soil samples for the other analyses were dried, crumbled, and sieved in 2 mm sieve. Saturation extracts obtained from the sieved soil samples were used to measure EC, pH, and temperature by using the EC/pH/T meter. The measured EC values at 25 °C were used to calculate the corrected EC_25_ values. By multiplying organic carbon contents with 1.724 organic matter contents of the samples were calculated [45]. Soil texture was determined by using the “Hydrometer Method” in [44] and described based on the USDA texture classification system.

Soil texture was sandy loam throughout the profile except the last layer which was sandy (Table 1). Bulk density was relatively high as expected for a sandy loam soil. Porosity changed with depth according to bulk density because it was calculated by using the bulk density-particle density relation with a constant particle density (2.65 g·cm^−3^ for mineral soils). Organic matter content was in intermediate level at the first two layers (20 cm tillage depth) but relatively low in the other layers as expected in a sandy loam soil due to the leaching. Saturated hydraulic conductivity of the soil increased with depth and was relatively high especially at the bottom depth corresponding to the sand content of the soil. The soil was in basic state with high pH. EC at 25 °C decreased with depth and was relatively low, as expected, due to the low clay content of the soil. The percent calcium carbonate (CaCO_3_) content of the soil was relatively high, possibly due to the high decomposition and low leaching processes in the soil (Table 1).

A salt (CaCl_2_) solution with a concentration of 3,200 mg·L^−1^ was applied by a rainfall simulator, having dimensions of 1.5 m by 1.0 m by 0.30 m, being made of an aluminum sheet with the thickness of 1 mm, and including 150 injectors (10 by 15 with 10 cm space) at the bottom of its reservoir which was 20 cm above the soil surface. Once, the solution concentration to be applied was determined as 3,200 mg·L^−1^ by considering the current soil EC, the applied water EC, and the maximum EC measuring value (4.5 dS·m^−1^) of the CS630 model TDR probes. Then the simulator was calibrated to obtain 2.962 and 4.060 cm·h^−1^ for the low and intermediate solution application rates by applying the same amount (12 cm) of solution within 4.051 and 2.956 h, respectively, in sandy loam soil.

When the infiltration capacity of the soil is less than the solution application rate, ponding occurs on the soil surface, resulting in preferential flow and transport automatically. The application rates were defined based on these considerations so that no ponding occured on the soil surface. However, the application durations and rates changed among the treatments even though the same amounts (12 cm) of solutions were applied for high application rate as flooding.

Although no ponding was observed on the soil surface for low and intermediate application rates during the experiments, ponding was inevitable in the treatments with high application rate as expected.

### 2.3. Experimental Treatments

The main factors, sub-factors and their interactions produced 12 (2 × 2 × 3) experimental treatments (Table 2). However, a total of 24 (2 × 2 × 3 × 2) plots was used in this study due to the design of the experiment based on the factorial block design with two replicates. While the undisturbed treatment was obtained by keeping the natural soil condition, the disturbed treatment was formed by digging the surface soil (1.5 m by 1.0 m) of the plot to a depth of 20 cm and replacing it after sieving through a 10 mm sieve. The dry initial SWC treatment was produced by keeping the natural soil water condition close to the permanent wilting point (SWC at soil water pressure of around 15 atm), whereas the wet initial SWC treatment was obtained by uniformly applying 100 L·m^−2^ of water to the plot surface before the experiement through a sprinkler attached hand-held plastic container. After the plot surface was covered by a plastic sheet to prevent evaporation, the plot was allowed to wait for two days for the initial SWC to reach about the field capacity (SWC at soil water pressure of around 0.33 atm). Low and intermediate solution application rates (2.962 and 4.060 cm·h^−1^, respectively) were determined based on no occurance of ponding on the soil surface using the rainfall simulator. The high solution application rate was obtained by simulating flood irrigation, where the solution was applied by a metal frame (1.5 m by 1.0 m by 0.25 m and the thickness of 1 mm).

### 2.4. Time Domain Reflectometry

The TDR100 (Campbell Scientific, Logan, UT, USA) was used in all experiments of this study. The TDR system comprised a TDR, datalogger and multiplexers. The TDR can be controlled by using either a datalogger or a computer with Windows software (PCTDR). While a datalogger with the TDR and multiplexers is used for automatic and multi-probes measurements, PCTDR is used for either manual measurements or setup and troubleshooting. A detailed theoretical background of TDR can be found in [46–48], but the main proceses including SWC and EC measurements in TDR is beriefly described as follows.

The apparent dielectric constant or relative permittivity of the medium (κ) can be used to calculate the volumetric SWC (θ) by using the universal calibration equation developed by [32] as:

(1) $$\theta =-5.3\times 10^{-2}+2.92\times 10^{-2}\kappa -5.5\times 10^{-4}\kappa^{2}+4.3\times 10^{-6}\kappa^{3}$$

The calibration equation (Equation (1)) has been confirmed by many authors including [49] for sandy to clay loam and [50] for sand and sandy loam with varying gravel contents. This equation can be safely used for estimation of θ in especially sandy soils with low clay content and thereby low influence of geochemistry as in this study. In [51] a procedure to determine soil bulk EC from TDR waveforms was proposed as:

(2) $$\sigma =\left(\frac{K}{Z_{u}}\right)\left(\frac{1-\rho_{\infty }}{1+\rho_{\infty }}\right)$$

where K is the geometric constant of a probe (m^−1^), Z_u_ is the characteristic impedance of a cable (Ω), and ρ_∞_ is the reflection coefficient (ρ_∞_ = (V_∞_ − V_0_)/V_0_), where V_∞_ is the signal amplitude at the distant point and V_0_ is the signal amplitude from the TDR instrument.

### 2.5. Conducting Experiments

**Figure 1.**
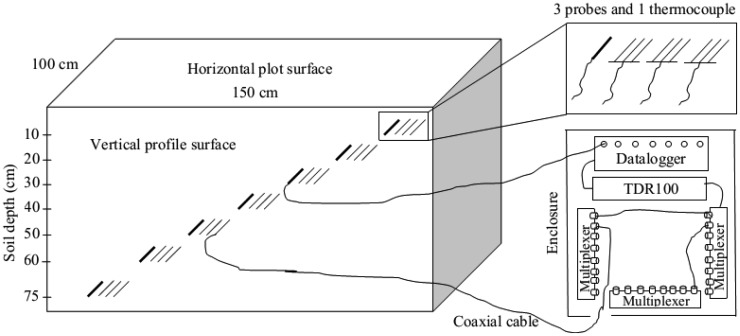
A sketch of the experimental design. Connection of a single thermocouple to the datalogger and connection of a single probe to the multiplexer are shown in the skecth to make presentation simple.

A soil profile was dug along 1.5 m of the experimental plot (1.5 m by 1.0 m) to be able to horizontally insert three CS630 model TDR probes (3-rod, 15 cm long, and 0.318 cm in diameter) for measuring SWC and EC in soil depths of 10, 20, 30, 40, 50, 60, and 75 cm using a probe insertion guide. A sketch of the experimental design for a plot of a treatment is illustrated in Figure 1. As seen in the figure, a total of 21 TDR probes and seven thermocouples (one for each depth) inserted to the soil looked like a 7-level stair starting from the surface right to the bottom left of the profile. This configuration was designed to prevent the negative effects of an upper probe on the lower probe due to the disturbance of upper soil during the probe insertion. The thermocouples inserted near the probes were used to measure soil temperature. After the configuration of the TDR system and then measuring the initial SWC and EC, a total of 12 cm CaCl_2_ solution (3,200 mg/L) was applied to the plot surface at a certain rate with the rainfall simulator. As soon as the solution application was stopped, the plot surface was covered by a plastic sheet to prevent evaporation from the plot. The measurements were continued around 1 day in each plot based on the assumption that flow reaches equilibrium in a sandy loam soil within around this time. Several parameters such as La/L for SWC, EC, soil temperature, and waveform were measured by the TDR for each probe every 15 min and stored in the datalogger automatically.

### 2.6. Measured Data

The TDR measured EC and especially SWC had some instabilities with time, resulting in the values having no physical sense such as the negative or >1 values of SWC. To resolve the problem, the main trends of SWC and EC with time were visually determined on graphs and then the outliers were removed. For a given depth by averaging three probe readings, the SWC and EC values were determined. The final SWC and EC values for a specific depth of a treatment were obtained by finding the average of the values of six probe readings (three probes and two replicates for a depth). In addition, soil bulk EC (BEC) readings of the TDR were corrected as:

(3) $${\text{EC}}_{25}=\text{BEC}[1+0.02(25-\text{Tsoil})]$$

where Tsoil is soil temperature (°C) around the related TDR probes measured by thermocouple because they were sensitive to solute concentration and temperature [52]. For each depth of each plot BTCs were developed out of the corrected data.

### 2.7. Introduction of Models

In MACRO model the total soil porosity is divided into macropore and micropore flow regions and each flow region has its own saturation rate, hydraulic conductivity, flux, and solute concentration [53]. One of the main advantages of the model is that the extent of preferential flow can be determined by running it for single- or dual-porosity approach separately using the same soil hydraulic properties. On the other hand, VS2DTI model uses the single-porosity or single-permeability or equilibrium modeling approach in which soil pores are assumed to be uniformly distributed throughout the soil, resulting in uniform flow [54]. The model can make either one-dimensional or two-dimensional simulations, but one-dimensional simulation mode of the model was used here to be able to compare the results with the results of one-dimensional MACRO model. The comparative description of the models is tabulated in Table 3.

Application of the models which have ability to simulate steady- and/or nonsteady-state flow and transport that requires different modeling approaches (single- or dual-permeability) helps the user to determine the extent of preferential or nonuniform flow and transport in a soil as in this Solute transport parameters can be determined by inversely fitting the one-dimensional CDE to the BTCs data obtained from the experimental measurements using the CXTFIT model developed by [59]. The model can also be used in the direct estimation (direct or forward modeling) of the transport parameters by utilizing the obtained parameter values. In the inverse estimation of solute transport parameters, the objective function (the sum of the squares of the differences between the measured and fitted data) is minimized. The transport scenerios used in the model are the conventional equilibrium CDE, the physical or chemical nonequilibrium CDE, and the stochastic stream tube model.

### 2.8. Parameterization of Models

MACRO and VS2DTI models were utilized to simulate SWC and EC measured along seven layer profiles of field sandy loam soil every 15 min during around 24 h using the TDR. The models had different parameter values for each soil layer because each layer had its own physical and hydraulic properties. Therefore, the determination of the model parameter values is explained as follows.

After solute transport parameters and measured physical and chemical properties of seven soil layers were input to MACRO, initial and boundary conditions and management parameters of the study area were set in the model. The constant hydraulic gradient was set as the bottom boundary condition. The management options such as no-tillage (for long-term), solution applied by irrigation, no drainage system and crop production in the study area, and non-reactive solute were selected in the model. The values of measured initial SWC and EC, solution application parameters (day of irrigation, amount of irrigation (120 mm), duration of irrigation (varying), and the concentration (3,200 mg/L) of solution applied by irrigation), and solute transport parameters were input to the model. The values of soil hydraulic parameters (θ_r_, θ_s_, α, n) used in the model were determined by fitting the [55] hydraulic function to the SWRC data by using the RETC programme [60]. Boundary soil water tension (CTEN) and corresponding boundary water content (XMPOR) values were determined on the SWRC visually. The values of saturated hydraulic conductivity (KSATMIN), boundary hydraulic conductivity (KSM), and the effective diffusion pathlength (ASCALE) were estimated by inputting the basic soil properties into the pedotransfer functions (PTFs; integrated into MACRO model [61–63]. The parameters used in the modeling with MACRO are presented in Table 4.

After the geometry and layer thicknesses of the soil profile were drawn by using the drawing tools in VS2DTI, the options of flow and transport model, iteration, and output were defined. Then the values of flow and transport parameters of each layer were determined. The values of soil hydraulic parameters (θ_r_, θ_s_, α, n) used in the model were determined by fitting the van Genuchten hydraulic function to the SWRC data using the RETC programme [60]. Saturated SWC values were obtained from SWRCs, whereas saturated hydraulic conductivity (Ks) values were determined by the PTFs integrated into MACRO model. Model parameters used in VS2DTI modeling studies are tabulated in Table 4. Initial SWC and EC values were input to the model as contour value on the drawn domain. Before running the model the boundary conditions were defined and the grid system of the domain was formed.

The options of the stochastic equilibrium CDE model and the field-scale resident concentration were set in the inverse modeling with CXTFIT. The applied solution concentration (3,200 mg·L^−1^) and its duration were determined after setting the option of pulse input at application time t as the boundary condition in the model. The measured initial concentrations were input to the model as initial condition after setting the constant initial concentration. The values of solute transport parameters (pore water velocity, v and dispersion coefficient, D) were obtained after inputting the measured BTC data and then running the model.

Bulk electrical conductivity (BEC) was measured directly by TDR as Siemens m^−1^ at different soil layers with time for the treatments. MACRO model produced the results for total solute storage in each layer as g·m^−3^ or ppm, whereas VS2DTI outputs for total solute storage at defined nodes were obtained as mg·cm^−3^. Then the solute storage outputs of MACRO and VS2DTI models were converted to the BEC to be able to compare with the BEC measured by TDR. The following equation was used in the conversion of model outputs to the BEC as [64]:

(4) $$\text{TDS}=640(\text{BEC})\text{for EC}<5\text{dS}\cdot {\text{m}}^{-1}$$

Therefore, BEC (assumed to be EC) was preferred for simulations because converting the TDR measurements and model outputs (in different units) to solute concentration, resulting in a 2-step conversion rather than a single-step conversion, increases uncertainty in the measurements and modeling.

### 2.9. Model Evaluation

The performances of the models in the estimation of the TDR measured SWC and EC were evaluated using some statistical parameters. The comparison of the means of SWC and EC values measured in the treatments was performed by Tukey test using the SPSS statistical programme as well as the means of the measured and estimated (by two models) values of SWC and EC in a treatment. Then they were grouped using the letters. In addition, the values of parameters like the coeficient of model efficiency (CME) and the root mean square error (RMSE) were computed for evaluation of the performances of the models (MACRO and VS2DTI) in estimation of SWC and EC in a soil depth of a treatment as:

(5) $$\text{CME}=\frac{{\sum_{\text{i}=1}^{\text{n}}\left({\text{O}}_{\text{i}}-{\text{O}}_{\text{m}}\right)}^{2}-\sum_{\text{i}=1}^{\text{n}}{\left({\text{P}}_{\text{i}}-{\text{O}}_{\text{m}}\right)}^{2}}{{\sum_{\text{i}=1}^{\text{n}}\left({\text{O}}_{\text{i}}-{\text{O}}_{\text{m}}\right)}^{2}}$$

(6) $$\text{RMSE}=\sqrt{\frac{{\sum_{\text{i}=1}^{\text{n}}\left({\text{O}}_{\text{i}}-{\text{P}}_{\text{i}}\right)}^{2}}{\text{n}}}$$

where O is the observed or measured, P is the model prediction or estimation, O_m_ is the mean of observed, and n is the sample number. The CME value changes between −∞ and +1 and it is aqual to 1 when the model perfectly estimates the measured data. RMSE can have any value between 0 and +∞. When the model performance is perfect, RMSE becomes zero, otherwise, it is larger than zero.

## 3. Results and Discussion

**Figure 2.**
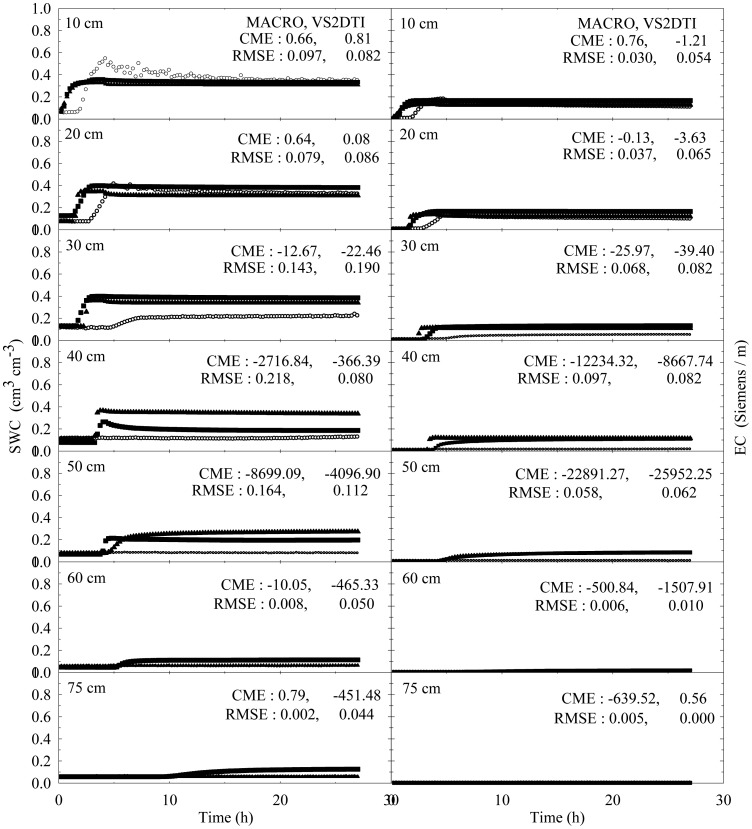
Spatial and temporal variability of soil water content (SWC) and electrical conductivity (EC) in the treatment of Sandy loam + Undisturbed + Dry + Low. The symbols of ○, ▲, and ■ represent the measured and model (MACRO and VS2DTI; values, respectively. CME and RMSE are the coeficient of model efficiency and the root mean square error, respectively.

**Figure 3.**
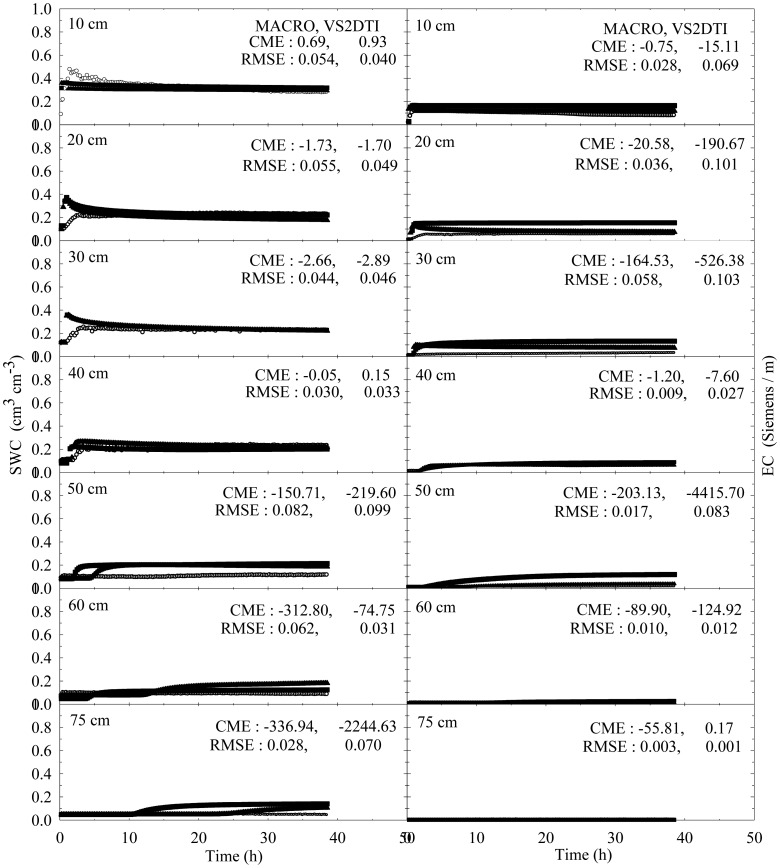
Spatial and temporal variability of soil water content (SWC) and electrical conductivity (EC) in the treatment of Sandy loam + Undisturbed + Dry + High. The symbols of ○, ▲, and ■ represent the measured and model (MACRO and VS2DTI; values, respectively. CME and RMSE are the coeficient of model efficiency and the root mean square error, respectively.

The experimental and modeling studies were conducted on the 12 treatments. The results of the spatial and temporal variation of the SWC and EC for the treatments 1 and 3 were visually presented in Figures 2 and 3 to illustrate flow and transport processes in an experimental plot.

**Figure 4.**
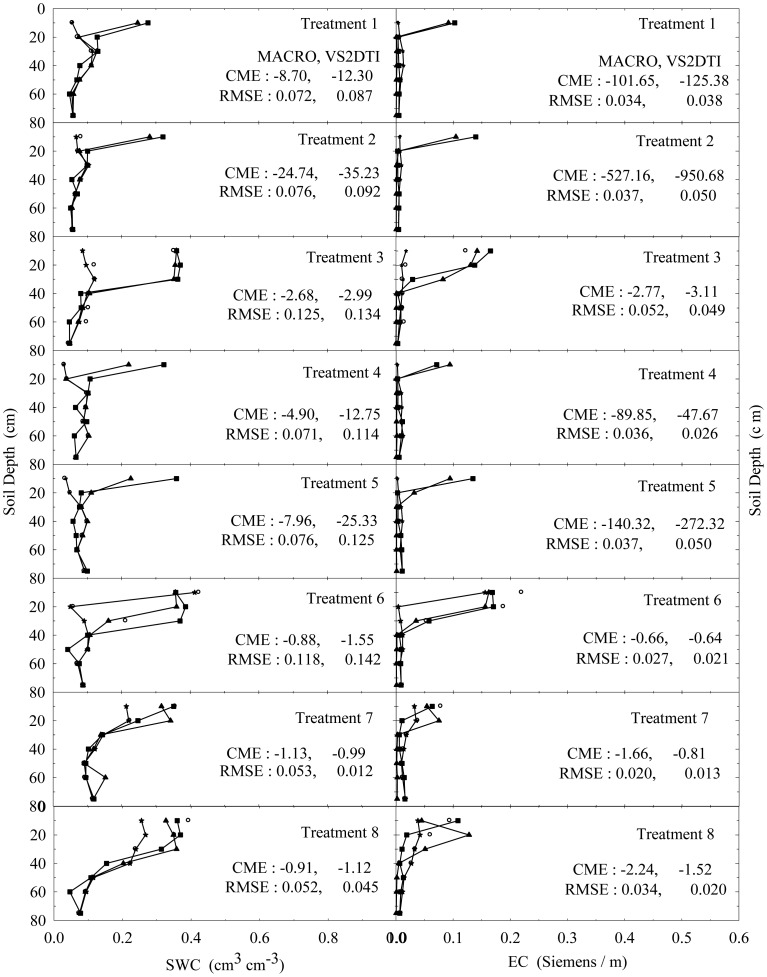
The variation of soil water content (SWC) and electrical conductivity (EC) with depth at 1 h of the experiment in the 8 treatments. The initial, measured, and model (MACRO and VS2DTI) values are exhibited by symbols ★, ○, ▲, and ■, respectively. CME and RMSE are the coeficient of model efficiency and the root mean square error, respectively.

**Figure 5.**
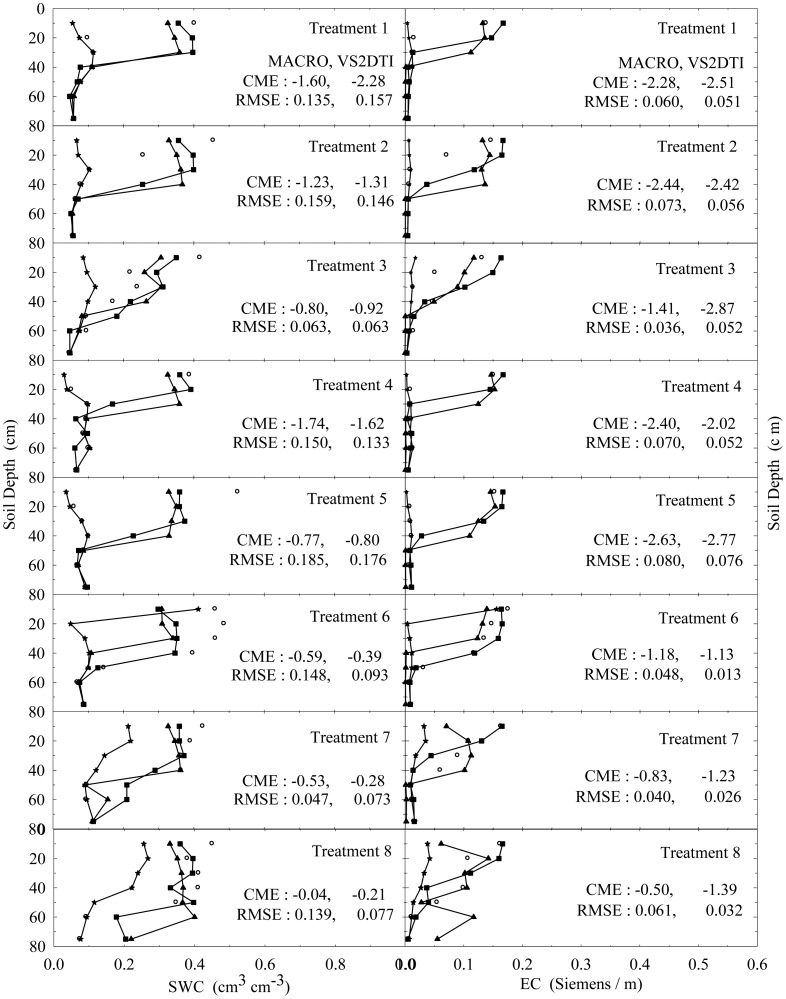
The variation of soil water content (SWC) and electrical conductivity (EC) with depth at 3 h of the experiment in the 8 treatments. The initial, measured, and model (MACRO and VS2DTI) values are exhibited by symbols ★, ○, ▲, and ■, respectively. CME and RMSE are the coeficient of model efficiency and the root mean square error, respectively.

**Figure 6.**
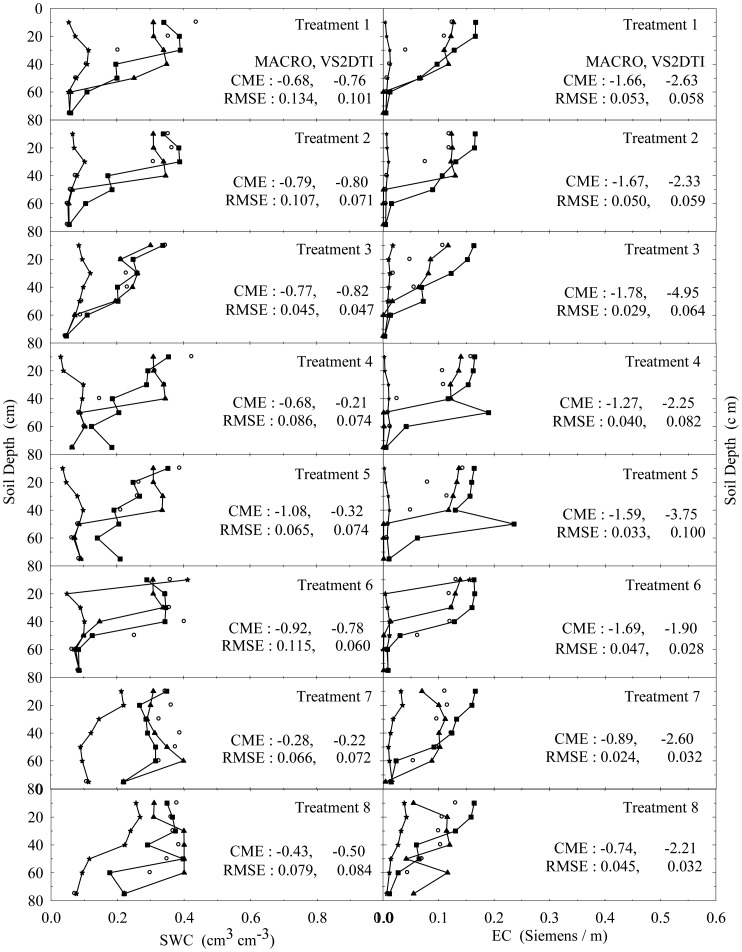
The variation of soil water content (SWC) and electrical conductivity (EC) with depth at 10 h of the experiment in the 8 treatments. The initial, measured, and model (MACRO and VS2DTI) values are exhibited by symbols ★, ○, ▲, and ■, respectively. CME and RMSE are the coeficient of model efficiency and the root mean square error, respectively.

In addition, the results of the only first 8 treatments (see Table 2) at 1, 3, and 10 h after the initiation of the experiments were also visually presented in Figures 4–6 in order to investigate the extent of preferential flow and transport. Not all results are presented due to the space limitation in the paper. However, the experimental and modeling results of all treatments were discussed in different details in the text and the results of statistical anaylses of all treatments were tabulated in Table 5.

SWC and EC changed until the 5th h of 30 cm of the 1st treatment (Figure 2). However, the applied solution increased SWC and EC up to the 1st h of 40 cm of the 3rd treatment (Figure 3). The initial SWC and EC did not change in the treatements 1, 2, 4, and 5 within the 1st h of the experiments (Figure 4). However, SWC and EC increased until 20 cm in the treatment 7, 30 cm in the treatments 8 and 10, 40 cm in the treatments 6 and 11, 50 cm in the treatments 9 and 11, and 75 cm in the treatment 3. In the 3rd h of the experiments, the applied solution changed SWC and EC up to various depths of the treatment plots (Figure 5). For instance, SWC and EC increased untill 30 cm in the treatments 1, 2, 4, and 5; 40 cm in the treatment 10; 50 cm in the treatments 7 and 11; 60 cm in the treatments 6, 8, 9, and 12; and 75 cm in the treatment 3. The applied solution moved further down in the profiles as the time passed and increased SWC and EC in 40 cm of the treatments 1 and 2; 50 cm in the treatments 4 and 5; 60 cm in the treatments 6, 10, 11, and 12; and 75 cm in the treatments 3, 7, 8, and 9 in the 10th h of the experiments (Figure 6).

As the initial SWC and EC did not change throughout the profiles of the treatments 1, 2, 4, and 5 in the 1st h of the experiments, they increased up to 40 and 50 cm with time in the treatments 1 and 2 and the treatments 4 and 5, respectively (Figures 4–6). The only difference between the treatments 1 and 4 and between the treatment 2 and 5 was that the surface 20 cm of the profiles were disturbed under dry initial SWC conditions. Similarly, the applied solution increased SWC and EC up to different depths with time at the treatments 7 and 10 and the treatments 8 and 11, where the only difference between the treatments 7 and 10 and between 8 and 11 was the disturbance of the soil profiles at the latter treatments under wet initial SWC conditions. Overall, even though there was no clear effect of soil structure on water flow and solute transport under dry and wet initial SWC conditions, it seemed to be somewhat more effective under wet initial SWC conditions than under dry initial SWC conditions. The applied solution moved slightly lower depths in the undisturbed soil profiles than in the disturbed profiles under the wet initial SWC conditions. These results confirmed the results of [65,66], where the applied solution moved deeper depths in structured (undisturbed) soils than in non-structured soils. In addition, [67] found that preferential flow in an Ap horizon with loamy sand was less than that of the underlying Bt horizon consisting of sandy clay to clay.

The increase in SWC and EC with time through the profiles of the treatments 7 and 8 was more than that of the treatments 1 and 2 under undisturbed soil conditions. Similarly, the applied solution increased SWC and EC with time through the profiles of the treatments 10 and 11 more than that of the treatments 4 and 5 under disturbed soil conditions. The results showed that the wet initial SWC was more effective on water and solute transport than the dry initial SWC condition and it caused water and solute to move deeper depths as reported by [19,20,68]. Besides, this effect of wet initial SWC condition was more obvious under the undisturbed soil condition compared to the disturbed condition.

As long as the other conditions were contant, when the treatments were compared for the effects of application rates among 4 treatment groups (treatments 1, 2, and 3; treatments 4, 5, and 6; treatments 7, 8, and 9; treatments 10, 11, and 12), the solution applied with the higher application rate moved lower depths at shorter durations in each group. Similarly, large rainfall events after herbicides application caused considerable amount of them to leach deeper depths in a sandy loam soil [23]. In addition, high application rate produced such a fast flow and transport as stated by [17,69]. Overall the results show that the applied solution moves in the soil somewhere between uniform and nonuniform or preferential flow and transport.

In the evaluation of the performances of the models (MACRO and VS2TI) for estimation of SWC and EC with space and time in a treatment, two statistical parameters (CME and RMSE) were calculated and the results were presented on Figures 2–6. However, only CME values were used in discussion of the model performances in the text because CME had the ability to evaluate both the comparative and absolute performances of a given model. VS2DTI model uses only single-porosity, or single-permeability, or equilibrium modeling approach, but MACRO models use both single-porosity and dual-porosity or nonuniform modeling approaches. However, the results of the only dual-porosity modeling approach were presented here because its results were slightly better than that of the single-porosity approach.

MACRO simulated SWC and EC better than VS2DTI in three of the seven depths and in six of the seven depths, respectively, in the 1st treatment (Figure 2), whereas it was better than VS2DTI in estimation of SWC in four depths and EC in five depths in the 3rd treatment (Figure 3). MACRO was better than VS2DTI in estimation of SWC and EC in seven and four out of the first eight treatments (see Table 2), respectively, in the 1st h of the experiments (Figure 4), but it simulated SWC and EC in 5 of the treatments better than VS2DTI in the 3rd h of the experiments (Figure 5). In addition, MACRO had better performance than VS2DTI in estimation of SWC and EC in four and all of the eight treatments, respectively, in the 10th h of the experiments (Figure 6). Although the performances of the models were relatively low in the estimation of SWC and EC with space and time, the results showed that MACRO had better performance than VS2DTI in estimation of SWC and especially EC. However, the superiority of MACRO to VS2DTI in estimation of SWC and EC was not so significant in especially some simulations. This suggests that both single- and dual-porosity modeling approach may be applied in the simulations of water and solute transport in sandy soils, where there was a balance between uniform and nonuniform or preferential flow and transport in the soil. On the other hand, the simulation performance of MACRO may be improved a bit more by using the dual-porosity modeling approach with inverse modeling to better determine multi-parameter values [70].

Soil hydraulic parameters are crucial input data in modeling studies on water flow and solute transport in the vadose zone; therefore, their accurate determination through either measurement techniques and/or predictive methods (*i.e.*, PTFs) is essential in modeling of flow and transport processes [71]. Since the measurement techniques are time-consuming and costly, PTFs have been widely applied for estimating soil hydraulic properties. [72] investigated the uncertainty in modeling root zone water dynamics with two different models (SWAP (soil-water-atmosphere-plant) and ALHyMUS) using Mualem-van Genuchten soil hydraulic parameters (θ_s_, θ_r_, α, n, and Ks) determined by five different methods: (i) laboratory measurements, (ii) field measurements, and (iii) three PTFs. Even though the results showed a wide range of soil hydraulic parameter values produced by the different methods, the model performances with different parameter sets derived by measurement techniques and PTFs were quite similar. For both models the best performances were obtained with sets of hydraulic parameters determined with PTFs. In addition, the differences in the model results with the same data set were attributed to the uncertainty in the modeling approach. Similarly, [73] showed that good modeling performances for soil water content were obtained with different PTFs. Furthermore, these results suggest that PTFs can be safely used in the modeling of water and solute transport in vadose zone if reliable or accurate data on soil hydraulic parameters are not available.

The model parameter values used in the modeling studies were presented in Table 4. The first 6 parameters were used in both models, but the last five parameters were used only in MACRO. Since clay or sand content of the soil was relatively constant throughout the profile, the values of the soil hydraulic function parameters (θ_r_, θ_s_, α, n) of [55] were also relatively constant throughout the profiles except the last depth, where sand content increased significantly. Ks values were high and stable along the profile except the 2nd depth, but dispersivity was constant throughout the profile. CTEN had a constant value of 20 along the profile, but the corresponding XMPOR value slightly increased with depth. KSM had relatively stable values along the profile except the last depth, where it was small, indicating that flow and transport through macropores were dominated as the sand content increased. Small and almost constant ASCALE values throughout the profile were expected in sandy soils with relatively small and homogeneous aggregate size or diffusion path-length. Tortuosity factor in macropores (ZN) was constant along the profile as expected in sandy soil with small ASCALE. [74] used the parameters XMPOR, KSM, ASCALE, and dispersivity with the values of 0.47 cm^3^·cm^−3^, 0.08 cm·h^−1^, 90 mm, and 3.5 cm, respectively, in their modeling study. The values of parameters CTEN, ZN, and dispersivity were 24.4 cm, 1, and 1 cm, respectively, in Lakeland sandy soil [75].

Tukey test was performed to analyze whether the differences among the means of the measured and simulated (by MACRO and VS2DTI models) SWC and EC in 12 treatments were statistically significant or not, and the results were presented in Table 5. The treatments were divided into three groups for measured and simulated (by MACRO, and VS2DTI) SWC, whereas they were divided into two, two, and four groups for measured, and MACRO and VS2DTI simulated EC, respectively. There were statistically significant differences among the groups in *p* < 0.01 level (Table 5). Even though all of the treatments were divided into the same number (=3) of groups for SWC, the measurements and simulations (by VS2DTI) produced different number of groups. In addition to the qualitative results presented in Figures 2–6, the quantitative statistical analyses also showed that there were significant differences among the treatments in terms of water and solute transport characteristics by being in different groups. If there were no statistically significant differences among the treatments in terms of SWC and EC, Tukey test would produce single group of the treatments.

Solute transport parameters (v and D) were inversely estimated by fitting the BTC data of the treatments to the one-dimensional CDE by using the CXTFIT program and the means of these parameter values were compared by the Tukey test. The results were presented in Table 5. The treatments were divided into different groups for both transport parameters (v and D) and there were statistically significant differences among the groups in p < 0.01 level. The differences among the treatments based on these two parameters were another indication of a certain degree nonuniform or preferential flow and transport in the soil [76,77].

## 4. Conclusions

In this study, individual and interactive effects of different factors like soil structure, initial SWC, and application rate on water flow and solute transport characteristices in a sandy loam field soil were studied by using the TDR measured SWC and EC, modeling the measured parameters (SWC and EC) with MACRO and VS2DTI models, and comparing the treatment means of SWC and EC and solute transport parameters by means of statistical analyses.

Overall the effects of soil structure on water flow and solute transport were not so clear under dry and wet initial SWC conditions, but the applied solution moved slightly lower depths in the undisturbed soil profiles than in the disturbed profiles under the wet initial SWC conditions. The applied solution moved lower depths in the wet initial SWC conditions than in the dry initial SWC condition and this effect of wet initial SWC condition was more distinctive under the undisturbed soil condition compared to the disturbed condition. The solutions applied with the higher application rates moved lower depths provided that the other conditions were contants, but it was difficult to differentiate the effects of the application rates on water and solute transport under different soil structural and initial SWC conditions. The interactive effects on water flow and solute transport may be better differentiated if more field experiments are conducted with the distinct interactive treatments.

Although the models had relatively low performances in the estimation of SWC and EC with space and time, the results showed that MACRO had somewhat better performance than VS2DTI. The differences among the 12 treatments determined using statistical analyses based on SWC, EC, and solute transport parameters were another indication of a certain degree nonuniform or preferential flow and transport in the soil.

## Figures and Tables

**Table 1.** Some physical and chemical properties of experimental field soil.

**Depth**	**Sand**	**Silt**	**Clay**	**Texture**	**BD**	**P**	**OM**	**Ks**	**pH**	**EC25**	**CaCO3**

(cm)	(%)	(%)	(%)		(g·cm^−3^)	(%)	(%)	(cm·h^−1^)		(dS·m^−1^)	(%)
10	77 (3.43) *	15 (2.95)	8 (0.62)	SL	1.54 (0.05)	42 (1.98)	1.25 (0.06)	2.292 (1.70)	7.89 (0.04)	0.688 (0.10)	24.61 (0.68)
20	78 (8.76)	14 (7.47)	8 (1.30)	SL	1.53 (0.07)	42 (2.71)	1.07 (0.08)	2.931 (0.77)	7.97 (0.12)	0.592 (0.11)	26.08 (1.39)
30	75 (2.56)	17 (1.53)	8 (1.20)	SL	1.52 (0.13)	43 (5.05)	0.86 (0.05)	2.759 (0.75)	8.00 (0.02)	0.556 (0.04)	28.62 (1.57)
40	72 (6.63)	20 (5.86)	8 (0.97)	SL	1.36 (0.04)	49 (1.50)	0.70 (0.02)	3.525 (0.48)	8.02 (0.02)	0.530 (0.08)	29.27 (0.59)
50	77 (3.52)	15 (4.11)	8 (1.53)	SL	1.39 (0.04)	48 (1.57)	0.69 (0.24)	5.942 (2.60)	7.98 (0.04)	0.492 (0.07)	30.69 (0.39)
60	77 (4.36)	15 (4.09)	8 (0.48)	SL	1.41 (0.05)	47 (1.86)	0.74 (0.19)	6.768 (3.92)	7.95 (0.05)	0.495 (0.02)	28.97 (3.18)
75	91 (1.63)	2 (1.80)	7 (0.59)	S	1.46 (0.04)	45 (1.54)	0.77 (0.10)	13.761 (4.74)	8.01 (0.11)	0.338 (0.09)	22.91 (5.64)

* The mean (standard deviation) of three replications, SL: Sandy loam, S: Sand, BD: Bulk density, P: Porosity, OM: Organic matter content, Ks: Saturated hydraulic conductivity, EC_25_: Electrical conductivity at 25 °C, and CaCO_3_: Calcium carbonate content.

**Table 2.** Experimental treatments.

**Treatment**	**Treatment**							

	**Soil Type Sandy loam**	**Soil Structure**		**Initial Soil Water Content**		**Solution Application Rate**		
		
		**Undisturbed**	**Disturbed**	**Dry**	**Wet**	**Low** *	**Intermediate**	**High** *
1	X	X		X		2.962		
2	X	X		X			4.060	
3	X	X		X				ponding
4	X		X	X		2.962		
5	X		X	X			4.060	
6	X		X	X				ponding
7	X	X			X	2.962		
8	X	X			X		4.060	
9	X	X			X			ponding
10	X		X		X	2.962		
11	X		X		X		4.060	
12	X		X		X			ponding

* Unit for application rate is cm h^−1^, ponding refers to flooding irrigation type of solution application.

**Table 3.** Comparison of model characteristics and/or abilities.

**Characteristic or ability**	**MACRO** [53]	**VS2DTI** [54]
Things to simulate	Water flow and movement of non-reactive solutes (Cl, Br), tritium, colloids, and pesticide	Water flow and solute and energy (heat) transport
State of flow and transport	Steady- and nonsteady-state	Steady-state or equilibrium
Dimension of simulation	One-dimensional	One- or two-dimensional
Domain	Macroporous layered soil	Gridded soil domain
Modeling approach	Single- or dual-permeability	Single-permeability
Governing equations	Richards Equation for water flow and CDE (convection-dispersion equation) for solute transport	Richards Equation for water flow and CDE (convection-dispersion equation) for solute transport
Soil hydraulic functions	Van Genuchten-Mualem [55,56]	Van Genuchten [55], Brooks and Corey [57], and Haverkamp [58]
Numerical technique to solve governing equations	Finite-difference	Finite-difference
Data requirement	Rainfall or irrigation and meteorological parameters (daily min. & max. air temperature, solar radiation, wind speed, and vapor pressure), initial and boundary conditions, and soil hydraulic functions	Geometry of domain, initial and boundary conditions, hydraulic and transport properties of porous medium, soil hydraulic functions, dispersivity, and molecular diffusion

**Table 4.** Model parameters used in the modeling studies.

**Depth (cm)**	**MACRO and VS2DTI**						**MACRO**				
	
	θr * (cm^3^·cm^−3^)	θs (cm^3^·cm^−3^)	α (cm^−1^)	n	Ks (cm·h&^minus;1^)	Disp. (cm)	CTEN (cm)	XMPOR (cm^3^·cm^−3^)	KSM (mm·h^−1^)	ASCALE (mm)	ZN
10	0.07088	0.36947	0.03932	1.64177	25.710	1	20	0.310	8.55	5	2
20	0.07046	0.41685	0.06441	1.61921	5.830	1	20	0.310	8.87	5	2
30	0.07708	0.39738	0.03396	1.70396	27.198	1	20	0.340	7.94	5	2
40	0.06478	0.46317	0.06360	1.56112	32.878	1	20	0.350	7.11	3	2
50	0.07102	0.42046	0.03711	1.75928	39.511	1	20	0.350	8.55	5	2
60	0.06848	0.43008	0.04336	1.65125	38.400	1	20	0.350	8.55	5	2
75	0.04660	0.23964	0.05583	1.56521	19.193	1	20	0.190	1.48	5	2

* θ_r_, θ_s_, α, n, Ks and l: Soil hydraulic function parameters of van Genuchten (1980) and Mualem (1976); θ_r_ and θ_s_: Residual and saturated SWC, respectively; α and n: Shape parameters of SWRC; Ks and l: Hydraulic conductivity parameters in Mualem (1976) function; Disp.: Dispersivity; CTEN: Boundary soil water tension; XMPOR and KSM: Boundary SWC and boundary hydraulic conductivity, respectively; ASCALE: Effective diffusion pathlength; and ZN: Tortuosity factor in macropores.

**Table 5.** Comparison of the means of the measured and simulated SWC and EC, and the solute transport parameters of the treatments using Tukey test.

**Treatment**	**Parameter**	**Measured**	**Model**		**Solute Transport Parameter**	
	
			**MACRO**	**VS2DTI**	**v (cm·h^−1^)** *	**D (cm2·h^−1^)**
1	SWC *	0.165^a^	0.227^c^	0.232^c^	17.22^abc^	72.29^abc^
	EC	0.040^a^	0.072^b^	0.085c		
2	SWC	0.170^a^	0.207^bc^	0.226^c^	41.16^abc^	500.00^d^
	EC	0.043^a^	0.068^b^	0.091^c^		
3	SWC	0.174^a^	0.193b	0.204^b^	41.16^abc^	500.00^d^
	EC	0.035^a^	0.052^b^	0.089^c^		
4	SWC	0.194^ab^	0.212^bc^	0.221^c^	21.33^abc^	357.57^abcd^
	EC	0.053^a^	0.069^b^	0.109^c^		
5	SWC	0.182^a^	0.211^bc^	0.221^c^	41.16^abc^	500.00^d^
	EC	0.052^a^	0.069^b^	0.119^d^		
6	SWC	0.255^c^	0.198^a^	0.230^b^	58.00^cd^	286.14^abcd^
	EC	0.077^b^	0.059^a^	0.094^c^		
7	SWC	0.296^ab^	0.303^b^	0.283^a^	37.76^abc^	78.16^abc^
	EC	0.080^a^	0.077^a^	0.092^b^		
8	SWC	0.304^b^	0.337^c^	0.302^b^	41.16^abc^	500.00^d^
	EC	0.076^a^	0.083^a^	0.082^a^		
9	SWC	0.288^b^	0.300^b^	0.299^b^	34.58^abc^	475.71^d^
	EC	0.064^a^	0.072^b^	0.099^d^		
10	SWC	0.294^a^	0.350^c^	0.305^ab^	27.34^abc^	79.15^abc^
	EC	0.073^a^	0.076^ab^	0.081^b^		
11	SWC	0.283^ab^	0.338^c^	0.268^a^	9.52^ab^	12.56^abc^
	EC	0.066^a^	0.081^b^	0.086^b^		
12	SWC	0.311^b^	0.314^b^	0.303^b^	53.31b^cd^	289.11^abcd^
	EC	0.091^b^	0.074^a^	0.091^b^		

* SWC: Soil water content, EC: Electrical conductivity, v: Pore water velocity, and D: Dispersion coefficient. The same letters; for a given parameter of Measured, MACRO, VS2DTI, v, or D; indicate the same groups of treatments, implying that there are no statistically significant differences among these groups of treatments in *p* < 0.01 level.
